# Evaluation of *Thymus vulgaris* and *Thymbra spicata* essential oils and plant extracts for chemical composition, antioxidant, and antimicrobial properties

**DOI:** 10.1002/fsn3.1007

**Published:** 2019-04-02

**Authors:** Ayça Gedikoğlu, Münevver Sökmen, Ayşe Çivit

**Affiliations:** ^1^ Department of Food Engineering, College of Engineering and Architecture Konya Food and Agriculture University Konya Turkey; ^2^ Department of Bioengineering, College of Engineering and Architecture Konya Food and Agriculture University Konya Turkey; ^3^ Strategic Research and Development Center Konya Food and Agriculture University Konya Turkey

**Keywords:** disk diffusion, FRAP, IC_50_, Microwave extraction, *Staphylococcus aureus*, thymol

## Abstract

The objectives of this study were (a) to obtain the essential oils (by hydrodistillation [HD] and microwave‐assisted extraction [MAE] methods) to determine the effect of the oil extraction method on the chemical composition, oil yield (%), free radical scavenging activity (IC_50_), ferric reducing antioxidant power (FRAP) value, and antimicrobial properties of *Thymus vulgaris* (thyme) and *Thymbra spicata* (zahter); and (b) to determine the effect of different solvents (methanol [80%] and ethanol [80%]) on extraction by means of the phenolic acid composition, total phenolic content, total flavonoid content, IC_50_, and FRAP value of thyme and zahter. Gas chromatography–mass spectrometry analysis showed that the amount of thymol (55.35%; 50.53%) and p‐cymene (11.2%; 11.79%) was found to be highest in thyme, when using HD and MAE, respectively. However, the highest amounts of carvacrol (68.20%; 66.91%) and γ‐terpinene (13.25%; 13.94%) were found in zahter, when using HD and MAE, respectively. Thyme essential oil had higher antioxidant capacity for both HD and MAE in comparison with zahter essential oil. Methanol extracts of both thyme and zahter had higher phenolic composition in comparison with their ethanol extracts. Extracts of both plants did not show any antimicrobial properties. However, essential oils of both thyme and zahter showed antimicrobial activity against chosen bacteria. Highest inhibition zone (radius) was shown against *Staphylococcus aureus *ATCC 9144 by the essential oils.

## 1 INTRODUCTION

*Thymus vulgaris* L. (thyme) and *Thymbra spicata* var. spicata (zahter) are aromatic herbs and members of the Lamiaceae family. These perennial shrubs are found in many parts of the world, especially in the Mediterranean region (Golmakani & Rezaei, 2008). Different varieties of Lamiaceae family plants have been found in various parts of Turkey (Kizil, Toncer, Diraz, & Karaman, 2015; Sokmen et al., 2004). Both thyme and zahter have a significant culinary use. Dry and fresh leaves of these herbs have been used extensively to flavor meat dishes, soups, and salads. In folk medicine, local people prepare herbal teas from these herbs to relieve headaches, toothaches, colds, asthma, and rheumatism (Lee, Umano, Shibamoto, & Lee, 2005; Uysal, Gencer, & Oksal, 2015).

Especially in last two decades, great efforts have been made to identify and quantify the bioactive components of Lamiaceae family plants. Essential oils of these plants are rich in thymol, carvacrol, p‐cymene, and γ‐terpinene (Golmakani & Rezaei, 2008; Lee et al., 2005; Sokmen et al., 2004; Tohidi, Rahimmalek, & Arzani, 2017). It has been reported based on in vitro tests that essential oils and their chemical constituents, such as thymol and carvacrol, provide antimicrobial and antioxidant properties (Sokmen et al., 2004; Uysal et al., 2015), which have been used in active packaging (Ballester‐Costa, Sendra, Fernandez‐Lopez, & Viuda‐Martos, 2016; Ramos, Beltran, Peltzer, Valente, & Garrigos, 2014; Ramos, Jimenez, Peltzer, & Garrigos, 2012) and in surface sanitation applications on fresh produce and meat (Lu, Joerger, & Wu, 2014; Lu & Wu, 2010,2012). In addition to the essential oils of these plants, hydrophilic fractions also have been studied. Research on extracts of L. family plant chemical has investigated their composition (Martins et al., 2015; Pereira, Peres, Silva, Domingues, & Cardoso, 2013) as well as their antioxidant (Martins et al., 2015; Skendi, Irakli, & Chatzopoulou, 2017) and antimicrobial properties (Erturk, Tanrikulu, Yavuz, Can, & Cakir, 2017; Fatma, Mouna, Mondher, & Ahmed, 2014).

Recently, the food industry has endeavored to move toward the direction of clean labeling. Therefore, removing synthetic preservatives, such as butylated hydroxytoluene (BHT) and butylated hydroxyanisole (BHA), from food formulations and replacing them with natural preservatives have gained significant importance. Many plants have been investigated for wide variety of active components for antimicrobial and antioxidant properties. Thyme and zahter could be useful plants due to their essential oils being high in thymol and carvacrol, respectively (Lee et al., 2005; Uysal et al., 2015), and also their extracts being rich in phenolic acids (Erturk et al., 2017; Roby, Sarhan, Selim, & Khalel, 2013). Phenolic acids, such as rosmarinic acid, have been studied for their antioxidant properties (Kostic et al., 2015; Skendi et al., 2017). Development of food additives made from essential oils and/or extracts with antioxidant and antimicrobial properties is a crucial step toward clean labeling and the production of healthier food products.

Few studies have investigated both the hydrophobic and the hydrophilic fractures of Lamiaceae family plants. This study provides a comprehensive investigation of both the essential oil and the plant extracts of two Lamiaceae family plants, *Thymus vulgaris *and *Thymbra spicata*. This study was designed to compare hydrophobic and hydrophilic extracts of both species. Hydrophobic fractions (essential oils) were obtained by hydrodistillation (HD) and microwave‐assisted extraction (MAE). MAE is fast and extracts essential oils quickly, so research is needed to determine whether oil compositions are affected by this extraction method. For the same reason, hydrophilic nonvolatile fractions will be extracted with two common solvents, methanol and ethanol. Therefore, the objectives of this study were (a) to obtain the essential oils by hydrodistillation (HD) and microwave‐assisted extraction (MAE) and to determine the effect of the oil extraction method on the chemical composition, oil yield (%), free radical scavenging activity (IC_50_), ferric reducing antioxidant power (FRAP) value, and antimicrobial properties; and (b) to determine the effect of different solvents (i.e., methanol [80%] and ethanol [80%]) on extraction by means of the phenolic acid composition, total phenolic content (TPC), total flavonoid content (TFC), IC_50_, FRAP values, and antimicrobial properties of thyme and zahter.

## 2 MATERIALS AND METHODS

### 2.1 Reagents and chemicals

High‐performance liquid chromatography (HPLC)‐grade methanol, acetonitrile, analytical grade ethanol, and GC‐grade hexanal were supplied by Merck KgaA (Darmstadt, Germany). Hydrochloric acid 37% was purchased from Macron Fine Chemicals (Gliwice, Poland). Additionally, 2,4,6 three(2‐pyridyl)‐S‐triazine (TPTZ) reagent AlCl_3_ anhydrous, 2,2‐diphenyl‐1‐picrylhydrazyl (DPPH), sodium bicarbonate anhydrous, gallic acid, and quercetin were supplied by Sigma‐Aldrich (Germany). Folin–Ciocalteu's phenol and sodium acetate trihydrate were supplied by Merck KgaA (Darmstadt, Germany). Iron(III) chloride hydrate and ferrous sulfate (FeSO_4_.7H_2_O) were purchased from Fisher Scientific (Leics, UK). Tryptic soy agar, tryptic soy broth, and Muller–Hinton agar were supplied by Merck KgaA (Darmstadt, Germany). Antimicrobial susceptibility test disks were purchased from Oxoid (Hants, UK).

### 2.2 Extraction of essential oil

#### 2.2.1 Microwave extraction

Air‐dried plants of cultivated *Thymus vulgaris *and *Thymbra spicata *were obtained from Diyarbakir Research Institute (July 2017), Turkey. A NEOS microwave extraction system (MA 125 Milestone, Italy) was used for this study. Seventy‐five grams of dry plant material was weighed into a beaker. Then, 150 ml of distilled water was added (1:2, w/v). After 30 min of resting, extraction was performed at 550 W power for 30 min. Then, the essential oil was collected and placed into vial with anhydrous sodium sulfate to remove any water. Then, vials were stored at 4°C for later analysis.

#### 2.2.2 Hydrodistillation with Clevenger apparatus

Hydrodistillation was conducted using an Electromantle™ (EM2000 CE, Electrothermal Engineering Ltd., UK, 500 W). Seventy‐five grams of air‐dried plant material was weighed into a round flask. Then, 750 ml of distilled water (1:10, w/v) was added. Extraction was performed for three hours. Next, the essential oil was collected and placed into a vial with anhydrous sodium sulfate to remove any water. Then, vials were stored at 4°C for later analysis.

### 2.3 Gas chromatography–mass spectrometry

Gas chromatography–mass spectrometry (GC‐MS) analysis of the essential oil was performed on a GC‐MS QP2020 equipped with an Rxi‐5Sil MS column (5% diphenyl‐95% dimethylpolysiloxane 30 m × 0.25 mm i.d., *df* = 0.25 µm; RESTEK GC Columns, USA). The injector and detector temperatures were set at 250°C. Temperature programming of the oven included an initial hold at 40°C for 2 min and a rise to 240°C at 4°C/min and held for 53 min. Helium was the carrier gas, with a linear velocity of 43.4 cm/s. The samples were diluted with *n*‐hexane (1:10, v/v), and a volume of 1.0 µl was injected into the GC with the injector in the split mode (split ratio: 1:25). The ionization voltage applied was 70 eV (electron volt), with a mass range *m*/*z *(mass‐to‐charge ratio) of 40–400 amu (atomic mass unit). The National Institute of Standards and Technology (NIST), WILEY 7 mass spectral library data, and authentic standards were applied to match the separated components. Peak area integration was used for the determination of percentage of combination. This analysis was carried out in duplicate.

### 2.4 Antioxidant assays

The 2,2‐diphenyl‐1‐picrylhydrazyl (DPPH) free radical scavenging capacity was measured according to Cuendet, Hostettmann, and Potterat (1997). Next, 50 µl of either essential oil or plant extract (at different concentrations in methanol) was mixed with 5 ml of a 0.004% (w/v) DPPH methanolic solution. The reaction was allowed to stand at room temperature for 30 min, and absorbance was read against a blank at 517 nm. The inhibitions of the DPPH radical in percent were calculated as follows: *I *(%) = (*A*_blank_ − *A*_sample_/*A*_blank_) × 100, where *A*_blank_ is the absorbance of the control reaction (containing all reagents except the test sample), and *A*_sample _is the absorbance value of the essential oil or the extract. Extract or oil concentration providing 50% inhibition (IC_50_) was calculated using the graph‐plotted inhibition percentage against the extract or oil concentration. Tests were conducted in duplicate, with gallic acid used as a positive control.

The ferric reducing antioxidant power (FRAP) assay was conducted according to Riahi et al. (2013). The FRAP reagent was freshly prepared by mixing acetate buffer (300 mM, pH 3.6), TPTZ solution (10 mM TPTZ in 40 mM HCl), and FeCl_3_·6H_2_O (20 mM) in a ratio of 10:1:1 (v:v:v). To carry out the assay, 900 µl of FRAP reagent, 90 µl distilled water, and 30 µl of plant extract or oil were mixed. After incubation at 37°C for 15 min, the absorbance was measured at 595 nm, using the FRAP solution as a blank. The antioxidant capacity of plant extracts or oil was determined from a standard curve plotted using the FeSO_4_·7H_2_O linear regression. Results were expressed as µM of Fe^+2^/g of essential oil or dry extract. Tests were conducted in duplicate.

### 2.5 Plant extraction and lyophilizing

Two solvents were used to extract polar fractions of the *Thymus vulgaris* and *Thymbra spicata*. Ten grams of sample was weighed into a beaker. Then, either 100 ml of 80% aqueous methanol (1:10 ratio of w/v) or 100 ml of 80% aqueous ethanol (1:10 ratio of w/v) was measured and placed into the beaker and shaken for 30 min to provide even mixing. Next, it was sonicated for 30 min, after which the slurry was filtered using a Buchner funnel, vacuum flask, and Whatman^®^ no. 1 filter paper. With the filtered solution in the dark and under refrigeration, the sonication procedure was repeated two more times. The collected filtrates were combined, and the liquid extract was placed into a 500‐ml round‐bottom flask. The aqueous methanol or aqueous ethanol solution was completely evaporated using a rotary evaporator at 50°C. Then, extracts were frozen at −80°C and freeze‐dried. The dried extracts were stored in the dark for later analysis. The proper amount of lyophilized extracts was dissolved in a 50% methanolic solution and filtered with a 0.45‐µm filter before use in the HPLC procedure.

### 2.6 HPLC analysis

#### 2.6.1 Standard preparation

A stock solution of standards was prepared at 1 mg/ml (1,000 ppm) concentration from a 50% methanolic solution. Working solutions of the standards were prepared at 1, 5, 25, 50, 75, and 100 ppm, and a 6‐point standard curve was prepared for each standard, based on the UV signal.

#### 2.6.2 HPLC conditions

A Waters model W2690/5 autosampler equipped with Waters 2,695 pumps and a Waters 2,489 UV detector was used for this study. Separation was achieved using an ACE C_18 _(5 µm − 4.6 × 250 mm) column (Advanced Chromatography Technologies Ltd., Aberdeen, Scotland). The mobile phases consisted of (A) 2% acetic acid, (B) acetonitrile and 0.5% acetic acid solution (1:1, v/v), and (C) acetonitrile. The column temperature was fixed at 25°C, and the injection volume was 20 µl. The flow rate was kept constant at 1.2 ml/min. A gradient program with the following proportions of solvents was used: We started the flow of mobile phase A as 95% and B as 5% to 5 min; this gradually increased to 20%, 22%, 25%, 27%, 40%, 45%, and 65% at 5, 8, 10, 17, 19, 30, 35, and 40 min, respectively, for mobile phase B, while mobile phase A decreased. Then, mobile phase B was reduced to 10% at 45 min, while mobile phase C increased to 90%. During the next 5 min, mobile phase C was 100%, followed by a return to the initial conditions for 10 min. The chromatographic peaks were identified based on their retention times and compared with the retention times of the authentic standards. Phenolic compounds found in the plants were calculated based on the external standard curve of the standard compounds. This analysis was carried out in duplicate.

### 2.7 Total phenolic and flavonoid content

To determine the total polyphenol content, 0.5 ml of the sample extract was mixed with 2 ml of Folin–Ciocalteu's reagent. After 5 min, 2.5 ml of 7.5% Na_2_CO_3 _solution was added, and the mixture was incubated for 90 min in the dark. The reaction mixture absorbance was measured at 760 nm, and the reaction mixture without the sample was used as a blank. Gallic acid was chosen as a standard, and a 6‐point standard curve was prepared (0–50 mg/L). The TPC of the plant extract was expressed as gallic acid equivalents (mg GA/g) for dry powder (Riahi et al., 2013; Singleton & Rosi, 1965). All samples were analyzed in duplicate.

To determine the TFC, 1 ml of diluted plant extract was mixed with 1 ml of 2% AlCl_3_ methanolic solution. After incubation at room temperature for 15 min, the absorbance of the reaction mixture was measured at 430 nm. Quercetin was chosen as a standard, and a standard curve was prepared (0–50 mg/L). The TFC was expressed as mg quercetin equivalents/g for dry weight (Djeridane et al., 2006; Riahi et al., 2013). All samples were analyzed in duplicate.

### 2.8 Antimicrobial activity

#### 2.8.1 Bacterial cultures

Three gram‐positive and three gram‐negative bacteria were used as test organisms: *Bacillus cereus* NRRL B3711, *Staphylococcus aureus* ATCC 9144, *Staphylococcus epidermidis* ATCC 12228, *Escherichia coli* ATCC 25922, *Salmonella enteritidis *ATCC 13076, and *Salmonella typhimurium *ATCC 14028.

#### 2.8.2 Disk diffusion assay

A disk diffusion assay was used to determine the antibacterial properties. Essential oils of hydrodistillation (HD) and microwave‐assisted extraction (MAE) along with methanol and ethanol extracts of *Thymus vulgaris *and *Thymbra spicata* were individually tested against six bacteria. All the bacterial species were first inoculated into tryptic soy agar and incubated overnight at 37°C. After checking for purity, the bacteria were suspended in a 0.9% NaCl solution using a densitometer to adjust the final cell concentration to a 0.5 McFarland number (1 × 10^8^ cfu/ml). Then, 100 µl of the bacterial suspensions was spread on Mueller–Hinton agar. The 6‐mm‐diameter, sterile, empty disks were either impregnated with 10 µl essential oils, or 20 µl of extracts was placed on the inoculated agar. Empty standard antibiotic disks were used as a control. The inoculated plates were incubated at 37°C for 24 hr. Antibacterial activity was determined by measuring the zone of inhibition in mm without including the radius of the disk.

### 2.9 Statistical analysis

All determinations were conducted in duplicate, and results for each parameter were expressed as the mean ± standard deviation. Data were evaluated by an analysis of variance (ANOVA) procedure. Means were separated by the least significant difference (LSD) when significant (*p* < 0.05) treatment effects were found.

## 3 RESULTS AND DISCUSSION

### 3.1 Essential oil yields

The total yields of volatile chemicals from *Thymus vulgaris *(thyme) and *Thymbra spicata *(zahter) are given in Table 1. Results showed that microwave‐assisted extraction (MAE) of zahter had a significantly (*p* < 0.05) higher oil yield (2.16% ± 0.16%) in comparison with its hydrodistillation (HD; 1.59% ± 0.06%). However, the distillation method did not affect the oil yield (%) for thyme (1.8 ± 0.14 for HD; 1.77 ± 0.05 for MAE). The essential oils of both thyme and zahter obtained with MAE were darker in color. Golmakani and Rezaei (2008) reported similar results for the essential oil of *Thymus vulgaris*: 2.52% for microwave‐assisted extraction, with the exception of a longer extraction time (2 hr) with a higher power wattage (990 W) and 2.39% for hydrodistillation (4 hr). Ozel, Gogus, and Lewis (2003) used supercritical water extraction for *Thymbra spicata* essential oil, finding that an increase in the extraction temperature also increased oil yield, but they found a comparable extraction efficiency of 2% at 100°C. Similar results were reported by Uysal et al. (2015) for the essential oil of *Thymbra spicata* using solvent‐free microwave extraction (2.5%) and hydrodistillation (2.4%). In another study, ultrasound‐assisted ohmic heating provided much higher oil yield (%) for *Thymus daenensis *in comparison with hydrodistillation (Tavakolpour et al., 2017). Microwave extraction provides comparable yields with other methods and uses less time, energy, and only a small amount of water to hydrate the dry plants.

**Table 1:** Yield of free radical scavenging activity and FRAP value of essential oils obtained by hydrodistillation (HD) and microwave‐assisted extraction (MAE) of *Thymus vulgaris *and* Thymbra spicata*

	*Thymus vulgaris* a		*Thymbra spicata* a	
	HD	MAE	HD	MAE
Yield (%, w/w)	1.8b ± 0.14	1.77b ± 0.05	1.59b ± 0.06	2.16a ± 0.16
DPPH^a^ (IC_50_, µg/ml)	159.59bc ± 12.79	93.77a ± 13.00	181.56c ± 2.13	129.48b ± 11.58
FRAP^a^ (µM Fe^+2^/g)	3.25a ± 0.017	3.18b ± 0.015	1.642c ± 0.0007	1.641c ± 0.0017

Note

DPPH: 2,2‐diphenyl‐1‐picrylhydrazyl; FRAP: ferric reducing antioxidant power.

Mean ±SD (*n* = 2).

a Different letters in the same row denote a significant difference, LSD Fisher's test (*p* < 0.05).

### 3.2 Antioxidant activity of the essential oils

To measure antioxidant activity, two assays—the DPPH (2,2‐diphenyl‐1‐picrylhydrazyl) free radical scavenging activity and the FRAP (ferric reducing antioxidant power)—were used to determine the in vitro antioxidant activities of the essential oils of *Thymus vulgaris *and *Thymbra spicata*. The free radical scavenging activity is higher for lower IC_50_ values. The amount of essential oil or extract needed to decrease the initial radical DPPH˙ concentration by 50% is used for the free radical scavenging activity and defined as IC_50_. Results of the antioxidant activity test are shown in Table 1. The free radical scavenging activity of thyme essential oil obtained with MAE (93.77 ± 13.0 µg/ml) was significantly (*p* < 0.05) higher than that found with HD (159.59 ± 12.79 µg/ml). When comparing the results with a corresponding commercial product (250 ± 0.01 µg/ml), the essential oil of thyme used in the current study had a much higher free radical scavenging activity (Teixeira et al., 2013). A similar pattern was observed for the free radical scavenging activity of the zahter essential oil. The MAE extraction (129.48 ± 11.58 µg/ml) had a significantly (*p* < 0.05) higher free radical scavenging activity than the HD (181.56 ± 2.13 µg/ml). Earlier studies found that compounds such as carvacrol and thymol exist in high amounts in Lamiaceae family plants. Lee et al. (2005) found that volatile components such as carvacrol and thymol had antioxidant properties that hindered the hexanal inhibition by 95%–100% over a 30‐day period at 5 µg/ml concentration. It was also reported that thymol and carvacrol were successfully used in active packaging as antioxidants (Ramos et al., 2014). The present study found that thyme had a higher antioxidant capacity than zahter. This could be due to the presence of a high thymol content in *T. vulgaris*. Previous studies found that thymol has a higher antioxidant activity due to the greater steric hindering effects of the phenolic group in thymol, which is greater than that of carvacrol (Yanishlieva, Marinova, Gordon, & Raneva, 1999), which corresponds with our results. Our study is the first to report on the determination of the DPPH˙ free radical scavenging activity of zahter essential oil. Both thyme and zahter essential oils obtained by HD and MAE had higher free radical scavenging activity in comparison with *Thymus spathulifolius *(0.243 ± 7.20 mg/ml; Sokmen et al., 2004), *Thymus mastichina* (3.11 ± 0.11 mg/ml), *Thymus zygis* (0.9 ± 0.03 mg/ml), *Thymus vulgaris* (4.05 ± 0.09 mg/ml), *Thymus capitatus* (0.6 ± 0.02 mg/ml; Ballester‐Costa, Sendra, Fernandez‐Lopez, Perez‐Alvarez, & Viuda‐Martos, 2017), and *Thymua danesis *subsp. *Lancifolius* (23.9 ± 0.6 mg/ml; Tavakolpour, Moosavi‐Nasab, Niakousari, & Haghighi‐Manesh, 2016). Similar results were also obtained in comparison with *Satureja thymbra *essential oil (96.7 µg/ml; Giweli, Dzamic, Sokovic, Ristic, & Marin, 2012).

Results of the ferric reducing antioxidant power (FRAP) are shown in Table 1. The essential oils of *Thymus vulgaris* have higher FRAP values in comparison with the essential oil of *Thymbra spicata*. The extraction method influenced the FRAP value of thyme but did not affect the FRAP values of zahter. Ballester‐Costa et al. (2017) reported Trolox equivalent FRAP values of different Thymus essential oils. They found that *T. capitatus* had the highest FRAP values, followed by *T. zygis*, *T. mastichina*, and *T. vulgaris*, respectively. Alizadeh, Alizadeh, Amari, and Zare (2013) reported FRAP values for *Thymus daenensis *of 24.23–26.45 µM quercetin equivalent/g for dry weight. In another study, the FRAP values of *Thymus mastichina *were studied for both essential oil and methanolic extracts, finding that due to the use of a phosphate buffer at pH 6.6, the essential oil underwent phase separation and did not produce proper results (Delgado et al., 2014). The literature has no FRAP values for zahter essential oil, making our results the first, to best of our knowledge. *Thymus vulgaris *(HD: 3.25 ± 0.017; MAE: 3.18 ± 0.015 µM Fe^+2^/g) and *Thymbra spicata *(HD: 1.642 ± 0.0007; MAE: 1.641 ± 0.0017 µM Fe^+2^/g) essential oils had significantly higher FRAP values than other species, such as the essential oil of *Artemisia absinthium* (0.595 ± 6.71 µM Fe^+2^/g; Riahi et al., 2013).

### 3.3 Chemical composition of the essential oils

Gas chromatography–mass spectrometry analysis resulted in the identification of 25 compounds for *Thymus vulgaris*, as shown in Table 2. A total of 97.2% of the oil obtained from HD and 94.35% of the oil obtained from MAE were identified, and any compounds found at 0.05% or less were not considered for identification. Thymol (55.3%; 50.53%), p*‐*cymene (11.2%; 11.79%), carvacrol (8.7%; 6.65%), and β‐caryophyllene (4.2%, 4.88%) were the main components of the essential oils when using HD and MAE, respectively. Also, there was no significant difference (*p* > 0.05) between the extraction procedures for these four major compounds. The other important compounds were γ‐terpinene (3.4%; 1.37%), isoborneol (2.3%; 2.79%), linalool (1.7%; 2.22%), and acetovanillone (1.7%; 4.55%) when using HD and MAE, respectively. A similar study conducted with Iranian *T. vulgaris* (Golmakani & Rezaei, 2008) reported lower thymol and carvacrol contents. Razzaghi‐Abyaneh et al. (2009) identified seven compounds in thyme and reported high thymol content (70.99%). Furthermore, Boruga et al. (2014) reported that the essential oil of thyme from Romania had lower thymol (47.59%) and p*‐*cymene (8.41%) levels but a much higher γ‐terpinene content (30.9%).

**Table 2:** Chemical composition of essential oils obtained by hydrodistillation (HD) and microwave‐assisted extraction (MAE) of *Thymus vulgaris*

No	Compound^c^	RI^b^	Relative peak area^a^ (%)	
			HD	MAE
1	α‐Thujene	824	0.5 ± 0.07	0.51 ± 0.35
2	α‐Pinene	829	0.4 ± 0.01	0.35 ± 0.21
3	Camphene	846	0.4 ± 0.02	0.45 ± 0.22
4	Vinyl amyl carbinol	880	0.8 ± 0.03	0.92 ± 0.01
5	Myrcene	889	0.7 ± 0.16	0.48 ± 0.17
6	2‐Ethylhexanol	898	0.2 ± 0.01	0.20 ± 0.01
7	α‐Terpinene	916	1.0 ± 0.23	0.92 ± 0.25
8	p‐Cymene	825	11.2 ± 1.89	11.79 ± 1.22
9	Limonene	928	0.4 ± 0.11	0.43 ± 0.08
10	γ‐Terpinene	960	3.4a ± 0.37	1.37b ± 0.19
11	Trans‐p‐menth‐2‐en‐1‐ol	969	0.8a ± 0.04	1.74b ± 0.04
12	Linalool	999	1.7a ± 0.07	2.22b ± 0.06
13	Isoborneol	1,070	2.3 ± 0.23	2.79 ± 0.25
14	Terpinen‐4‐ol	1,079	1.1 ± 0.30	0.79 ± 0.08
15	α‐Terpineol	1,109	0.1 ± 0.01	0.18 ± 0.06
16	Thymol	1,193	55.3 ± 1.2	50.53 ± 1.36
17	Carvacrol	1,214	8.7 ± 3.03	6.65 ± 2.10
18	β‐Caryophyllene	1,322	4.2 ± 0.18	4.88 ± 0.98
19	Aromadendrene	1,339	0.3 ± 0.01	0.39 ± 0.10
20	α‐Humulene	1,356	0.1 ± 0.01	0.15 ± 0.02
21	Viridiflorene	1,391	0.2 ± 0.01	0.29 ± 0.00
22	Δ‐Cadinene	1,417	0.2 ± 0.01	0.20 ± 0.06
23	Acetovanillone	1,460	1.7a ± 0.09	4.55b ± 0.21
24	Spathulenol	1,478	0.7 ± 0.06	0.67 ± 0.19
25	Caryophyllene oxide	1,483	0.9 ± 0.08	0.97 ± 0.27
Total peak area (%)			97.2	94.35

a Mean ± *SD* (*n* = 2).

b Retention Index on nonpolar HP‐5ms column in reference to *n*‐alkanes.

c Different letters in the same row denote significant difference, LSD Fisher's test (*p* < 0.05).

Gas chromatography–mass spectrometry analysis resulted in the identification of 20 compounds for *Thymbra spicata*, as shown in Table 3. All the constituents of the essential oil were not significantly (*p* > 0.05) different when using HD and MAE. Major components of the essential oil were carvacrol (68.20%, 66.91%), γ‐terpinene (13.25%, 13.94%), p*‐*cymene (5.37%, 4.65%), β‐caryophyllene (2.59%, 4.02%), and thymol (1.19%, 2.16%) when using HD and MAE, respectively. Similar results have been reported in several studies: Kizil et al. (2015) identified 23 compounds in zahter essential oil and found the carvacrol content to be 67.08% from the same region, while in another study, lower carvacrol content was reported, with 36.1% when using HD and 44.8% when using MAE for essential oils of zahter from Antalya, Turkey (Kizil et al., 2015; Uysal et al., 2015). Much higher carvacrol content (75.74%) was also found in zahter essential oil from Mugla, Turkey (Sarac, Ugur, & Duru, 2009). Inan, Kirpik, Kaya, and Kirici (2011) investigated various harvesting times (i.e., before flowering, during flowering, and after flowering) on the essential oil constituents of zahter. Results of their study showed that γ‐terpinene decreased after flowering, while carvacrol increased. Similarly, Barakat, Wakim, Apostolides, Srour, and Beyrouthy (2013) found differences in the content of the constituents of the essential oil of zahter from Lebanon obtained before, during, and after flowering. They also reported the highest p*‐*cymene content (8.1%–46.8%) in comparison with our study and other studies from Turkey. Research has shown that many factors involving extraction parameters, such as temperature, time, and type of extraction procedure as well as location, soil composition, moisture, altitude, and many other environmental factors can influence the content and composition of an essential oil. Our study demonstrated that the microwave extraction method gives similar results in comparison with the traditional Clevenger method.

**Table 3:** Chemical composition of essential oils obtained by hydrodistillation (HD) and microwave‐assisted extraction (MAE) of *Thymbra spicata*

No	Compound	RI^b^	Relative peak area^a^ (%)	
			HD	MAE
1	α‐Thujene	824	1.05 ± 0.47	0.25 ± 0.21
2	α‐Pinene	829	0.44 ± 0.17	0.11 ± 0.07
3	Hepten‐3‐ol	880	0.3 ± 0.04	0.26 ± 0.14
4	Myrcene	889	1.58 ± 0.42	0.84 ± 0.32
5	2‐Ethylhexanol	898	0.11 ± 0.01	0.23 ± 0.23
6	α‐Phellandrene	904	0.19 ± 0.04	0.12 ± 0.04
7	α‐Terpinene	916	1.89 ± 0.38	1.29 ± 0.18
8	p‐Cymene	825	5.37 ± 1.11	4.65 ± 1.28
9	Limonene	928	0.23 ± 0.06	0.23 ± 0.08
10	γ‐Terpinene	960	13.25 ± 1.41	13.94 ± 4.12
11	Linalool	999	0.15 ± 0.00	0.14 ± 0.01
12	Isoborneol	1,070	0.13 ± 0.01	0.12 ± 0.04
13	Terpinen‐4‐ol	1,079	0.47 ± 0.09	0.25 ± 0.02
14	Thymol	1,193	1.19 ± 0.05	2.16 ± 0.59
15	Carvacrol	1,214	68.20 ± 3.57	66.91 ± 7.52
16	β‐Caryophyllene	1,322	2.59 ± 0.38	4.02 ± 0.99
17	Aromadendrene	1,339	0.21 ± 0.04	0.37 ± 0.08
18	α‐Humulene	1,356	0.09 ± 0.01	0.13 ± 0.03
19	Spathulenol	1,478	0.2 ± 0.03	0.37 ± 0.18
20	Caryophyllene oxide	1,483	0.46 ± 0.01	0.32 ± 0.06
Total peak area (%)			98.06	96.66

a Mean ± *SD* (*n* = 2).

b Retention Index on nonpolar HP‐5ms column in reference to *n*‐alkanes.

Different letters in the same row denote a significant difference, LSD Fisher's test (*p* < 0.05).

### 3.4 Chemical compositions of the plant extracts

The chemical composition of the *Thymus vulgaris *and *Thymbra spicata *methanol and ethanol extracts is shown in Table 4. Both of the plant extracts had higher phenolic acids for a majority of the compounds prepared with 80% methanol extract in comparison with 80% ethanol extract. Rosmarinic acid, benzoic acid, rutin, gallic acid, and cinnamic acid were the only compounds for which both the extraction solvent and type of plant had a significant (*p* < 0.05) effect on the content. Rosmarinic acid was found highest in zahter, with 15.65% in the methanol extract in comparison with 7.65% in the ethanol extract, while the rosmarinic acid content for thyme with the methanol extract was 13.66% and with the ethanol extract, 8.69%. High rosmarinic acid content has a great potential because of its possible antioxidant, antibacterial, and antiviral properties (Kostic et al., 2015). Our results agree with other studies reported for members of Lamiaceae family plants (Martins et al., 2015; Roby et al., 2013). Some researchers reported lower rosmarinic acid content (Delgado et al., 2014; Skendi et al., 2017). Erturk et al. (2017) studied *Ocimum bacillicum *(sweet basil) and *Thymbra spicata* from Amasya, Turkey. They did not report rosmarinic acid content, but the highest percentages of phenolics were found to be trans‐cinnamic acid (0.0545%) and syringic acid (0.0183%). We found a cinnamic acid content of 0.037% (methanol) and 0.021% (ethanol) for thyme and 0.026% (methanol) and 0.011% (ethanol) for zahter. Roby et al. (2013) reported very high cinnamic acid content, with 28.54% for thyme. Many factors could influence the phenolic composition of the plants, such as the extraction solvent, methodology, location, soil composition, season, altitude (Kizil et al., 2015; Magwaza et al., 2016; Roby et al., 2013). Among the identified and quantified compounds, the second most frequently occurring compound after rosmarinic acid was benzoic acid. Its content ranged from 0.53% to 0.75% for zahter and 1.08% to 1.47% for thyme*, *when using ethanol and methanol extracts, respectively. Benzoic acid is an aromatic carboxylic acid naturally found in plant and animal tissues. It can be produced by the plant as protection against fungal attacks. Benzoic acid and its derivatives have been employed in the food industry as antifungal and antibacterial food additives (Olmo, Calzada, & Nunez, 2017). Based on all the available data, we found that *Thymus vulgaris* and *Thymbra spicata* provided a relatively high amount of rosmarinic acid and benzoic acid. Plant extracts with such high levels of rosmarinic acid and benzoic acid can be used as a natural food additive to improve the shelf life of food products.

**Table 4:** Composition of phenolic compounds, total phenolic content (TPC), total flavonoid content (TFC), free radical scavenging activity, and FRAP value of *Thymus vulgaris *and *Thymbra spicata *obtained by methanolic and ethanolic extraction

Phenolic compound (%)	Approximate	*Thymus vulgaris* a		*Thymbra spicata* a	
	Rt (min)	80% Methanol	80% Ethanol	80% Methanol	80% Ethanol
Gallic acid	4.78	0.251a ± 0.051	0.167ab ± 0.006	0.059bc ± 0.028	0.032c ± 0.009
4‐Hydroxybenzoic acid	13.209	0.159 ± 0.047	0.085 ± 0.026	0.091 ± 0.016	0.059 ± 0.012
Chlorogenic acid	13.890	0.017 ± 0.007	0.015 ± 0.003	0.072 ± 0.061	0.117 ± 0.002
Syringic acid	17.276	0.062 ± 0.012	0.043 ± 0.007	0.08 ± 0.02	0.054 ± 0.017
Coumaric acid	23.204	0.051 ± 0.008	0.051 ± 0.007	0.053 ± 0.001	0.052 ± 0.001
Rutin	28.021	0.353a ± 0.100	0.325a ± 0.142	0.987b ± 0.075	1.053b ± 0.106
Benzoic acid	31.398	1.471a ± 0.155	1.077ab ± 0.102	0.752bc ± 0.027	0.532c ± 0.078
Cinnamic acid	33.710	0.037a ± 0.003	0.021ab ± 0.001	0.026ab ± 0.011	0.011b ± 0.001
Rosmarinic acid	37.617	13.66b ± 0.145	8.696c ± 0.055	15.645a ± 0.001	7.653d ± 0.009
Quercetin	43.067	0.988 ± 0.402	0.619 ± 0.378	0.599 ± 0.278	0.252 ± 0.019
TPC (mg GAE/g DW)		15.13a ± 0.313	13.57b ± 0.103	13.14b ± 0.135	13.13b ± 0.249
TFC (mg QUE/g DW)		7.285a ± 0.021	6.17b ± 0.100	4.36c ± 0.069	3.24d ± 0.058
DPPH^a^ (IC_50_, µg/ml)		29.22a ± 0.385	36.77b ± 0.45	35.28b ± 0.45	43.9c ± 0.845
FRAP^a^ (µM Fe^+2^/g)		30.88a ± 0.02	26.93b ± 0.025	14.99c ± 0.015	14.19d ± 0.01

Note

DPPH: 2,2‐diphenyl‐1‐picrylhydrazyl; FRAP: ferric reducing antioxidant power.

Mean ± *SD* (*n* = 2).

a Different letters in the same row denote a significant difference, LSD Fisher's test (*p* < 0.05).

### 3.5 Total phenolic content and total flavonoid content of the extracts

The results of the TPC of the plant extracts are shown in Table 4. The *Thymus vulgaris *methanol extract had significantly (*p* < 0.05) higher TPC than its 80% ethanol extract and also more than the *Thymbra spicata* methanol and ethanol extracts. The TPC ranged between 13.13 and 15.13 mg GAE/g for dry weight. Sokmen et al. (2004) reported very high TPC at 141 mg GAE/g DW for *T. spathulifolius*. However, Skendi et al. (2017) reported between 34.3 and 70.4 mg GAE/g DW of TPC for Lamiaceae family plant methanol extracts. In contrast, Fatma et al. (2014) reported 7.05–8.81 mg GAE/g DW for *T. hirtus *sp. algeriensis from various locations in Tunisia. Roby et al. (2013) reported levels of TPC from 4.75 to 8.10 mg GAE/g DW for *T. vulgaris*. Our results align with these reported studies. The TFC results are displayed in Table 4. The TFC ranged from 3.24 to 7.285 mg QUE/g DW for all the extracts. Methanol extracts exhibited significantly (*p* < 0.05) higher amounts of TFC in comparison with ethanol extracts. Also, thyme extracts had a significantly (*p* < 0.05) higher amount of TFC than zahter extracts. Tohidi et al. (2017) reported between 1.89 and 8.55 mg QUE/g among the 14 Thymus species from Iran and found the highest TFC in *T. vulgaris*, with 8.55 mg QUE/g. However, in some other studies, the TFC was determined based on rutin equivalent (Fatma et al., 2014; Miliauskas, Venskutonis, & van Beek, 2004). Both the TPC and the TFC supplied information about the antioxidant capacity because higher phenolic content is associated with higher antioxidant activity.

### 3.6 Antioxidant activity of the extracts

Reactive oxidative species, such as singlet oxygen, hydroxyl radical, peroxyl radical, and lipid hydrogen peroxide, cause oxidation of food products. Food oxidation can lead to the production of toxic compounds as well as a loss of nutrition. Also, oxidation reduces the shelf life and consumer acceptability of food products. Antioxidants scavenge radicals in food by donating hydrogen to these radicals and produce antioxidant radicals with low reduction potential. Also, due to the low reduction potential of these antioxidant radicals, they cannot cause oxidation of other molecules (Choe & Min, 2009). Phenolic compounds found in plants are great examples of natural antioxidants and can be used as food additives in formulations, coatings, or active packaging, to slow the oxidation of food products. Results of the antioxidant tests are shown in Table 4. The free radical scavenging activity (IC_50_ value) of the methanol extracts of thyme and zahter (29.22 ± 0.385; 35.28 ± 0.45) was significantly (*p* < 0.05) higher than the ethanol extracts of these plants (36.77 ± 0.45; 43.9 ± 0.845), respectively. Similar results were reported by Lagouri, Bantouna, and Stathopoulos (2010). The difference in the DPPH radical scavenging activity might result from the extraction method, extraction solvent, geographical location, or difference in plants within the same family. These variations were reported in previous studies (Fatma et al., 2014; Martins et al., 2015; Roby et al., 2013; Skendi et al., 2017).

Based on the obtained results, the thyme plant extracts had significantly (*p* < 0.05) higher antioxidant capacity than the zahter plant extracts. We also determined the IC_50_ value of gallic acid 1.74 µg/ml as a positive standard. Plant extracts provided lower antioxidant capacity in comparison with pure gallic acid. Results of the ferric reducing antioxidant power (FRAP) assay are shown in Table 4. The FRAP value of the thyme plant extracts was significantly (*p* < 0.05) higher than that of the zahter plant extracts. FRAP values were between 14.19 and 30.88 µM Fe^+2^/g. In addition, methanol provided significantly (*p* < 0.05) higher FRAP values in comparison with ethanol extracts. The results of the DPPH and FRAP assays were highly correlated. Similar results were observed by Ballester‐Costa et al. (2017). Comparing the results with Table 1 shows that hydrophilic fractions have higher radical scavenging activity and FRAP values than the essential oils of these plants. This might be explained by the fact that the bioactive compounds of essential oils are more effective in inhibiting conjugated dienes than free radical scavenging activity. Sokmen et al. (2004) found similar results for *Thymus spathulifolius*.

### 3.7 Antibacterial activity of the extracts

Results of the antibacterial activity test based on disk diffusion are shown in Table 5. The antibacterial activity of *Thymus vulgaris *and *Thymbra spicata *essential oil and plant extracts was tested against gram‐positive and gram‐negative bacteria. Plant extracts did not display any antibacterial effects against the tested bacteria, while essential oils of both plants showed an antibacterial effect. The maximum effect was found against *Staphylococcus aureus* ATCC 9144 (24–35 mm), and the minimum activity was shown against *Salmonella typhimurium* ATCC 14028 (11–14.5 mm). The essential oil of *Thymus vulgaris *that underwent microwave‐assisted extraction displayed significantly (*p* < 0.05) higher antibacterial activity against four bacteria than did the hydrodistilled essential oil. The major chemical components of the essential oil, such as thymol and carvacrol, have been associated with antimicrobial properties (Rota, Herrera, Martinez, Sotomayor, & Jordan, 2008; Sokmen et al., 2004). This also aligns with our results. However, the fact that these major components occurred in a much higher quantity in hydrodistilled essential oil (Tables 1 and 2) indicates that minor components can also influence antibacterial properties. Similar results were reported earlier (Bounatirou et al., 2007; Rota et al., 2008). For *Thymbra spicata*, the microwave‐assisted extraction of essential oil provided a larger inhibition zone (23–35 mm) for Gr (+) bacteria, while hydrodistilled oil provided a larger inhibition zone (14–19 mm) for Gr (−) bacteria. Overall, both essential oils showed a higher inhibition effect against Gr (+) bacteria in comparison with Gr (−) bacteria; this could be due to the difference in the wall type for Gr (+) and Gr (−) bacteria (Erturk et al., 2017; Teixeira et al., 2013). In addition to successful results obtained from in vitro antimicrobial studies, thyme essential oil or its bioactive components such as thymol and carvacrol have been successfully used as antimicrobial agents in surface washes on fresh produce and meat (Lu et al., 2014; Lu & Wu, 2010,2012). The essential oil of both *Thymus vulgaris *and *Thymbra spicata *provided an antibacterial effect against these six bacteria. This is especially important because these bacteria are major foodborne pathogens (except *E. coli* ATCC 25922) associated with serious foodborne illnesses. Therefore, the essential oils of *Thymus vulgaris *and *Thymbra spicata *can be useful, natural antimicrobials for food preservation.

**Table 5:** Antibacterial activity of essential oils obtained by hydrodistillation (HD) and microwave‐assisted extraction (MAE) of *Thymus vulgaris *and* Thymbra spicata* tested using the disk diffusion method (mm)

Bacterial species	*Thymus vulgaris* a (mm)		*Thymbra spicata* a (mm)	
	HD^a^	MAE^a^	HD^a^	MAE^a^
*Bacillus cereus* NRRL B3711	27b ± 1.12	31.5a ± 1.30	24.5b ± 1.48	24.5b ± 0.83
*Staphylococcus aureus* ATCC 9144	24.5c ± 2.96	29b ± 2.24	24c ± 1.42	35a ± 1.00
*Staphylococcus epidermidis* ATCC 12228	27b ± 1.00	30a ± 1.42	21c ± 2.24	23c ± 1.00
*Escherichia coli* ATCC 25922	14.5b ± 0.87	15b ± 1.00	19a ± 2.24	13b ± 2.24
*Salmonella enteritidis* ATCC 13076	13.5b ± 0.87	21.5a ± 1.66	19a ± 1.42	14.5b ± 0.87
*Salmonella typhimurium* ATCC 14028	13.5a ± 0.87	14.5a ± 1.66	14a ± 1.42	11b ± 1.00

Mean ± *SD* (*n* = 2).

a Different letters in the same row denote a significant difference, LSD Fisher's test (*p* < 0.05).

## 4 CONCLUSION

This study provided a comprehensive investigation of hydrophilic and hydrophobic fractures of *Thymus vulgaris *and *Thymbra spicata *for chemical composition, antioxidant activity, and antimicrobial activity. Concerning the extraction methodology for essential oils, microwave‐assisted extraction provided results comparable to hydrodistillation for the GC‐MS and antioxidant assays. To the best of our knowledge, the results of the FRAP assay for *Thymbra spicata *essential oil are the first to be reported. Bioactive compounds, such as rosmarinic acid, were high in both methanol extracts of the plants. Also, methanol extracts provided significantly (*p* < 0.05) higher bioactive compounds than ethanol extracts, which also relates to their antioxidant activity, TPC, and TFC. Plant extracts did not provide antimicrobial activity; on the other hand, essential oils showed antimicrobial activity. Use of both extracts and essential oil in emulsions could provide antioxidant and antimicrobial activity for food preservation. Future studies should be performed to determine whether extracts and essential oils in emulsions show a synergistic relationship for antioxidant and antimicrobial properties for food products.

## ETHICAL STATEMENT

This study does not involve any human or animal testing.

## CONFLICT OF INTEREST

The authors have no conflict of interest to declare.
